# Case Report: Successful Stabilization of Marburg Variant Multiple Sclerosis With Ocrelizumab Following High-Dose Cyclophosphamide Rescue

**DOI:** 10.3389/fneur.2021.696807

**Published:** 2021-06-23

**Authors:** Valeria Koska, Moritz Förster, Katja Brouzou, Maryam Hatami, Ercan Arat, Ahmet Aytulun, Philipp Albrecht, Orhan Aktas, Patrick Küry, Sven G. Meuth, David Kremer

**Affiliations:** Department of Neurology, Medical Faculty, Heinrich-Heine-University, Düsseldorf, Germany

**Keywords:** Marburg MS, malign MS, high dose cyclophosphamide, ocrelizumab, neurofilament

## Abstract

The Marburg variant of multiple sclerosis (Marburg MS) is the most aggressive form of MS, often leading to death soon after onset. Here we describe the case of a 26-year-old Marburg MS patient presenting with severe neurological deficits requiring intensive care. In spite of more than 100 gadolinium-enhancing MRI lesions, the patient recovered almost completely upon high-dose cyclophosphamide (HiCy) rescue treatment (four consecutive days with 50 mg/kg/day, cumulative absolute dose of 14 g). Following the acute treatment, her disease was stabilized by B cell depletion using ocrelizumab. Clinical amelioration was reflected by a decrease of MRI activity and a marked decline of serum neurofilament light chain levels. HiCy rescue treatment followed by ocrelizumab as a maintenance therapy prevented permanent disability and achieved an almost complete clinical and drastic radiological improvement in this Marburg MS patient.

## Introduction

The Marburg variant of multiple sclerosis [Marburg MS (1)] which accounts for <4% of the total incidence of MS cases mostly affects children and young adults (2). It is a fulminant form of MS, featuring an acute onset of severe neurological deficits often resulting in death within weeks to months (3). Histology usually shows extensive demyelination as well as necrosis, which often involves vital areas like the brainstem (3). Synonyms for Marburg MS, which is the most aggressive variant of the disease, include malign, acute fulminant, acute malignant, and rapidly progressive MS. Its most important differential diagnoses are acute disseminated encephalomyelitis, Balo's concentric sclerosis, and Schilder's diffuse sclerosis. Treatment has proven to be challenging, but recent reports documented positive outcomes following an administration of high-dose cyclophosphamide (4). Here we describe the case of a 26-year-old Marburg MS patient initially presenting with symptoms of a bilateral optic neuritis and no further abnormalities upon examination. In this case, we successfully used ocrelizumab as a maintenance therapy following a high-dose cyclophosphamide induction protocol and monitored the therapy response by serum neurofilament light chain (NfL) levels.

## Case Presentation

**Figure 1:**
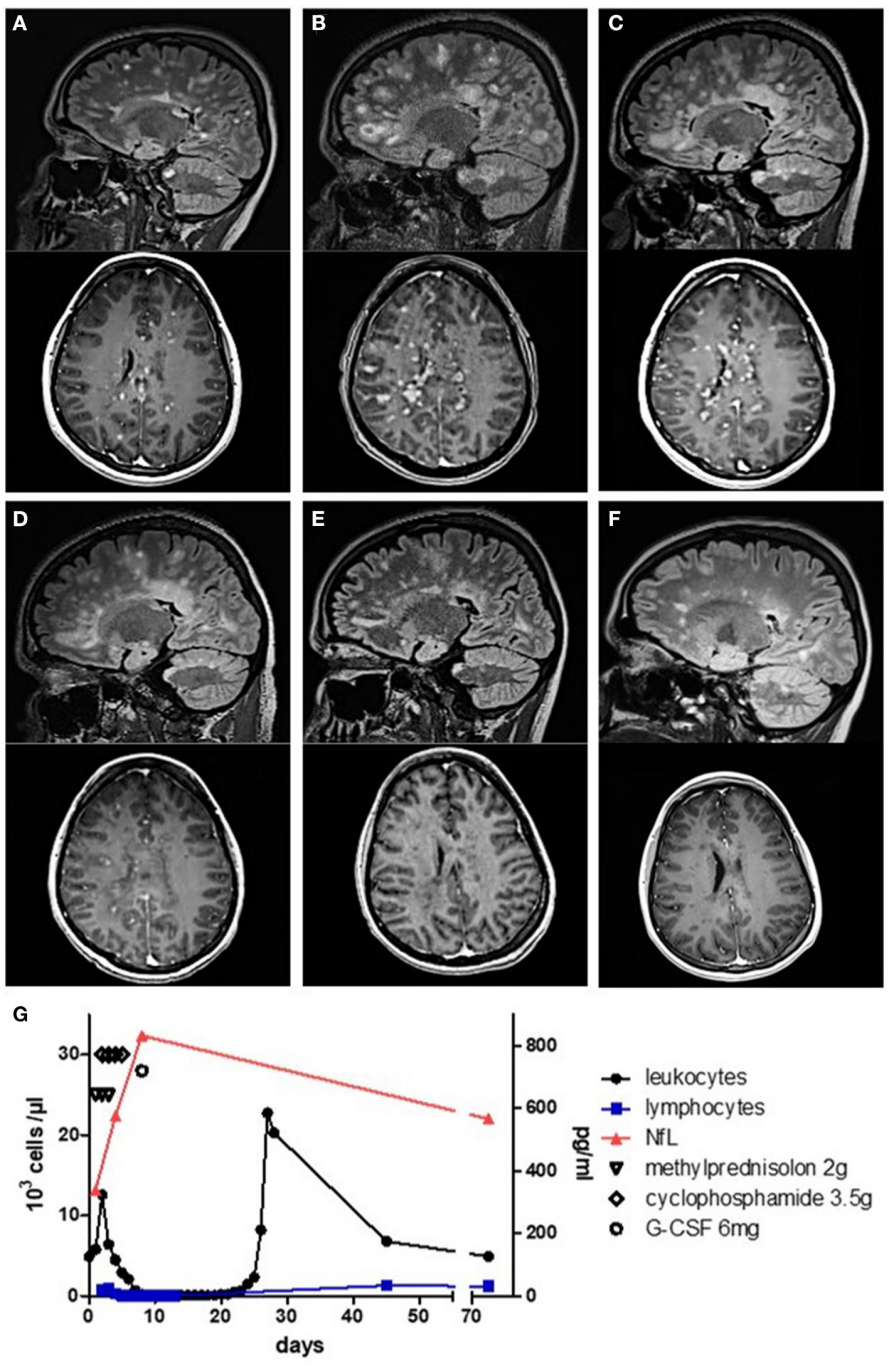
MRI and serological monitoring of the disease course. **(A–F)** Sagittal FLAIR and axial T1 gadolinium (Gad) sequences. **(A)** Numerous lesions with Gad enhancement at first admission. **(B)** MRI at re-admission showing more than 100 Gad-enhancing lesions. **(C)** Decrease of lesion load 7 days after the first dose of cyclophosphamide. **(D)** Further decrease of lesion load mirroring clinical remission at 15 days and **(E)** at 30 days. **(F)** At 44 days after cyclophosphamide treatment, no more Gad enhancement was detectable. **(G)** Leukocyte and lymphocyte count as well as serological neurofilament light chain levels during treatment.

A 26-year-old female patient without a history of neurological symptoms presented to our hospital with bilateral optic neuritis which had gradually developed within 5 days prior to admission. Other than hypothyreosis, her past medical history was unremarkable. The patient complained of mainly left-sided bilateral blurred vision, reduced color discrimination, and pain with eye movement. Visual acuity was impaired on both eyes (left: 20/80; right: 20/40). Ophthalmological examination yielded no retinal abnormalities, but optic coherence tomography (OCT) was not possible as the patient was unable to focus appropriately. She was unable to read letters bilaterally during low-contrast visual acuity (LCVA) testing using 2.5% low-contrast Snellen charts. Visual evoked potentials (VEPs) yielded no response for the left eye, while P100 latencies for the right eye were pathologically increased to 139.5 ms. At admission, neurological examination revealed no further abnormalities. Cerebrospinal fluid (CSF) analysis 9 days after the first occurrence of symptoms revealed a lymphomonocytic pleocytosis of 72/μl with positive oligoclonal bands (OCBs) and intrathecal IgM synthesis. The MRZ reaction (MRZR), anti-AQP4, and anti-MOG antibodies as well as vasculitis screening and virological/microbiological analyses were all negative (see Table 1). 1 day later, intravenous corticosteroids (1,000 mg/day for five consecutive days) were initiated, and on the following day, numerous supra- and infratentorial gadolinium (Gad)-enhancing and non-enhancing T2w as well as two Gad-enhancing spinal lesions consistent with the diagnosis of multiple sclerosis (MS) were seen on MRI (Figure 1A). In detail, there was Gad enhancement in both optic nerves, more than 35 dot-shaped Gad-enhancing juxtacortical/periventricular lesions with typical Dawson's finger configuration, and more than 12 active infratentorial lesions. Due to the typical presentation and the above-described paraclinical findings, we dispensed with further imaging such as magnetic resonance spectroscopy or positron emission tomography–computed tomography. As intravenous steroids resulted in no clinical improvement, five cycles of plasmapheresis and two additional cycles of immunoadsorption were performed. Following this treatment, her sight ameliorated subjectively as she regained the ability to recognize outlines, but overall improvement was poor. While LCVA yielded a score of 0 bilaterally, OCT was now possible, showing a normal peripapillary retinal nerve fiber layer thickness in both eyes (right: 102 μm; left: 103 μm). The patient was scheduled for ocrelizumab therapy within the following 2 weeks and discharged. Regarding other therapeutic options, we decided against alemtuzumab due to her known diagnosis of hypothyroidism. Moreover, the patient was opposed to natalizumab treatment due to concerns regarding progressive multifocal leukoencephalopathy even though she had a negative anti-John–Cunningham virus antibody titer. 1 day after discharge, the patient received the 13-valent pneumococcal conjugate vaccine in preparation for the planned ocrelizumab treatment. Within the next 5 days (i.e., ca. 1 month after the initial symptoms), she experienced a second relapse and was re-admitted to our hospital with a newly developed left spastic hemiplegia and progressive loss of vigilance. MRI revealed a fulminant progression of the lesion load and Gad enhancement with now more than 100 Gad-enhancing lesions (Figure 1B). CSF analysis prior to the following therapy showed a shift to a lymphogranulocytic pleocytosis. Serum NfL was 340 pg/ml as measured by ELISA (Figure 1G). After exclusion of an infectious etiology, a course of intravenous high-dosage corticosteroid (2,000 mg/day for 3 consecutive days) was administered. On the second day of corticosteroid therapy, the patient was started on an additional high-dose cyclophosphamide (HiCy) therapy for 4 consecutive days with 50 mg/kg/day, reaching a cumulative absolute dose of 14 g. Shortly after this combined therapy, serum NfL peaked at 833 pg/ml. 3 days after HiCy therapy, stem cells were mobilized with 6 mg granulocyte-colony stimulating factor. As expected, blood analysis revealed leukopenia and lymphopenia immediately after HiCy treatment. A recovery of the leukocytic population was observed at 10 days later. Circa 3 days after the last dose of cyclophosphamide, we observed both clinical and radiological improvement. While the total lesion load was stable, only 16 lesions were still active (Figure 1C). Peripheral CD34-positive hematopoietic stem cells (HSC) were harvested by leukapheresis 3 weeks later and cryopreserved for future transplantation, if needed. The patient was discharged with mild residual neurological deficits and was closely monitored both clinically and radiologically (Figures 1D–F). Serum NfL levels slowly decreased over the course of 10 weeks to 566 pg/ml. 9 weeks after the HiCy treatment, maintenance therapy with ocrelizumab was initiated following standard dosing (i.e., loading with 300 mg i.v. twice within 2 weeks and 600 mg i.v. as maintenance). 6 months later, the patient was still clinically stable without any relapses. The only residual symptom was a slightly impaired visual acuity (left: 20/30; right: 20/30). The VEP P100 latencies had returned to normal (right, 110 ms; left, 109 ms).

**Table 1:** Laboratory characteristics and differential diagnoses.

**Characteristics**	**OCBs**	**Positive**	**Cranial lesions**	**Yes**	**Spinal lesions**	**Yes**
	**MRZ**	**Negative**	**Anti-AQP4-IgG**	**Negative**	**Anti-MOG-IgG**	**Negative**
Differential diagnoses	Vitamin B1, B6, B12, folic acid	Normal	ANA, ANCA, ENA	Negative	ACE	16 U/l
	Vitamin D	8 ng/ml	JCV-PCR	Negative	JCV titer	0
	Anti-HIV 1/2 IgG	Negative	Borrelia and treponema IgG/IgM	Negative	Toxoplasmosis-PCR	Negative
	Anti-HHV6 IgG	Positive	Cryptococcosis antigen	Negative	Anti-toxoplasmosis IgG/IgM	Negative
	Anti-*Candida* antibodies	Negative	Anti-*Aspergillus* antibodies	Negative	Bartonella IgG/IgM	Negative

*OCBs, oligoclonal bands; ANA, antinuclear antibodies; ANCA, anti-neutrophil cytoplasmatic antibodies; ENA, extractable nuclear antigen antibodies; ACE, angiotensin-converting enzyme; JCV, John–Cunningham virus*.

## Discussion

The initial manifestation of MS in our patient raised several red flags, suggesting a severe disease course, such as persisting and disabling symptoms, almost no recovery after the first steroid pulse, and a high lesion load at baseline, including spinal and infratentorial Gad-enhancing lesions. In addition, intrathecal IgM synthesis is associated with an unfavorable prognosis (5). Thus, following an acute relapse treatment, we aimed at a highly effective immunodepletion therapy with ocrelizumab. However, when the mostly fatal Marburg variant of MS became evident, we were forced to take a more aggressive therapeutic approach. This decision was made since, of the 28 cases documented in the literature, only 11 (39%) survived, of whom only two (18%) had a favorable outcome. Of note is that the Marburg MS is still poorly defined as an MS variant in its own right as demonstrated by the heterogeneous nomenclature found in the literature (e.g., malign MS, acute fulminant MS, acute malignant MS, rapidly progressive MS, etc.). As a result, the clinician is faced with uncertainty regarding the potential transferability of previous therapeutic approaches. While there are cases in which mitoxantrone or alemtuzumab were successfully applied (6, 7), we advocate the use of cyclophosphamide as this agent not only effectively targets immune cells but also mobilizes HSC. These can then be harvested for later therapeutic use in the form of autologous hematopoietic stem cell transplantation, which has proven to be a potent therapy for highly active relapsing–remitting MS (8) and might therefore be effective as a long-term therapy in Marburg MS as well. Furthermore, following the high-dose cyclophosphamide protocol of Krishnan et al. (4) and using ocrelizumab as a maintenance therapy, we were able to prevent permanent disability and to achieve an almost complete clinical and drastic radiological improvement. As most other reported cases were fatal, this will provide the unique opportunity to monitor the long-term disease course. Regarding the pharmacodynamics of our treatment, we did not measure the cyclophosphamide levels in our patient. However, a case series of seven patients from 1,983 compared cyclophosphamide levels in the serum and CSF of MS patients following oral administration and showed identical levels, which demonstrated the high CNS penetration of this medication (9). As our patient had a reduced level of consciousness, we decided for intravenous administration. So far, there have been two case reports with favorable outcomes using the protocol that we applied (4, 10). Of note is that the cyclophosphamide treatment was primarily intended as a rescue therapy for the fulminant disease course and not as a means of stem cell mobilization. In general, the doses needed for effective immunosuppression are far higher than those required for stem cell mobilization (11). Beyond the Marburg MS cases, the dose that we applied was also evaluated in refractory MS patients (12). Regarding side effects, high doses of cyclophosphamide can induce hemorrhagic cystitis, hepatic damage, and cardiac necrosis (13) as well as infertility and ovarian endocrine failure (14). However, neither during the acute treatment phase nor during follow-up for 6 months were any of these side effects observed. Concerning ocrelizumab, this medication depletes CD20-positive B and T cells (15) within days but may take up to 3 months to reach its maximal effect. However, upon consultation with the patient, we decided to use it as a maintenance therapy due to her concerns described above. With regard to disease biomarkers, serum NfL levels in MS reflect ongoing disease activity and correlate with worsening of disability, lesion load (16), and risk for relapses. Accordingly, we found a rapid increase of NfL levels in our patient which doubled within a period of 7 days, reflecting the fulminant disease course. On the other hand, our data suggest that NfL may also be a suitable tool to monitor therapy response in Marburg MS, as we found it to steadily decrease following the HiCy treatment. To our knowledge, this is the first case of Marburg MS where data on this serological biomarker of CNS damage were collected during disease and recovery. Another interesting feature of our case was that, while we found positive OCBs, the MRZ reaction was negative. In MS, the MRZR, a polyspecific antiviral immune response against measles, rubella, and varicella zoster virus has a specificity of ~97% (17). Unfortunately, the majority of the other Marburg MS case reports did not include information on this laboratory marker so that, at present, its significance in Marburg MS remains unclear. Lastly, it is an important feature of this case that the severe disease reactivation occurred shortly after anti-pneumococcus vaccination. Of note is that there have been recent studies describing a worsening of MS after the administration of live attenuated vaccines like yellow fever (18), but inactivated vaccines, like pneumococcal vaccination, are usually considered safe regarding MS activity. In summary, we trust that this report will contribute to a more successful management of Marburg MS.

## Conclusion

In this case, we were able to prevent permanent disability and to achieve an almost complete clinical and drastic radiological improvement using ocrelizumab as a maintenance therapy following the high-dose cyclophosphamide protocol of Krishnan et al. NfL may be a suitable tool to monitor therapy response in Marburg MS.

## Data Availability Statement

The raw data supporting the conclusions of this article will be made available by the authors, without undue reservation.

## Ethics Statement

Ethical review and approval was not required for the study on human participants in accordance with the local legislation and institutional requirements. The patients/participants provided their written informed consent to participate in this study. Written informed consent was obtained from the individual(s) for the publication of any potentially identifiable images or data included in this article.

## Author Contributions

VK, SM, and DK gave the idea of case reporting. VK and DK analyzed the case and prepared the MRI scans as well as the figure and the table. VK drafted the manuscript for intellectual content. MF, KB, MH, EA, AA, PA, OA, PK, SM, and DK critically reviewed the manuscript and were involved in patients' healthcare. All the authors contributed to the article and approved the submitted version.

## Conflict of Interest

PA received compensation for serving on scientific advisory boards for Allergan, Biogen, Celgene, Ipsen, Merck Serono, Merz Pharmaceuticals, Novartis, and Roche. He received speaker honoraria and travel support from Allergan, Bayer Vital GmbH, Biogen, Celgene, Ipsen, Merck Serono, Merz Pharmaceuticals, Novartis, and Roche and research support from Allergan, Biogen, Celgene, Ipsen, Merck Serono, Merz Pharmaceuticals, Novartis, and Roche. OA has received grant support from Bayer, Biogen, Novartis, and Sanofi and consultancy or speaking fees and fees for serving on steering committees from Bayer, Biogen, Celgene, Medimmune, Merck, Novartis, Roche, Sanofi, and Teva. PK was supported by Stifterverband/Novartisstiftung. SM received honoraria for lecturing and travel expenses for attending meetings from Almirall, Amicus Therapeutics Germany, Bayer Health Care, Biogen, Celgene, Diamed, Genzyme, MedDay Pharmaceuticals, Merck Serono, Novartis, Novo Nordisk, ONO Pharma, Roche, Sanofi-Aventis, Chugai Pharma, QuintilesIMS, and Teva. His research was funded by the German Ministry for Education and Research (BMBF), Deutsche Forschungsgemeinschaft (DFG), Else Kröner Fresenius Foundation, German Academic Exchange Service, Hertie Foundation, Interdisciplinary Center for Clinical Studies (IZKF) Muenster, German Foundation Neurology, Almirall, Amicus Therapeutics Germany, Biogen, Diamed, Fresenius Medical Care, Genzyme, Merck Serono, Novartis, ONO Pharma, Roche, and Teva. DK received travel grants from GeNeuro and Merck, refund of congress participation fees from GeNeuro, Merck, and Servier, consulting fees from Grifols, payment for lectures from Grifols, and support for research projects from Teva and was funded by the Deutsche Forschungsgemeinschaft (DFG) while carrying research on human endogenous retroviruses at Cleveland Clinic. The remaining authors declare that the research was conducted in the absence of any commercial or financial relationships that could be construed as a potential conflict of interest.
